# Dysfunctional Metacognitive Beliefs in Patients With Obsessive–Compulsive Disorder and Pattern of Their Changes Following a 3-Month Treatment

**DOI:** 10.3389/fpsyt.2021.628985

**Published:** 2021-04-22

**Authors:** Shin Tae Kim, Chun Il Park, Hae Won Kim, Sumoa Jeon, Jee In Kang, Se Joo Kim

**Affiliations:** ^1^Department of Psychiatry, Yonsei University College of Medicine, Seoul, South Korea; ^2^Institute of Behavioral Science in Medicine, Yonsei University College of Medicine, Seoul, South Korea; ^3^Department of Psychiatry, CHA Bundang Medical Center, CHA University, Seongnam, South Korea; ^4^Department of Medical Education, Yonsei University College of Medicine, Seoul, South Korea

**Keywords:** dysfunctional metacognitive beliefs, metacognition, negative beliefs about worry, OCD, treatment response

## Abstract

**Introduction:** Metacognitions are considered to be crucial factors for the development and maintenance of pathologic anxiety. The present case–control study aimed to examine how metacognitive beliefs are associated with the diagnostic status and subtypes of obsessive–compulsive disorder (OCD). In addition, we examined the pattern of changes in metacognitive beliefs after a 3-month pharmacological treatment in patients with OCD.

**Methods:** A total of 562 cases with OCD and 236 healthy controls were assessed with the Metacognitions Questionnaire (MCQ) and various measures of OC symptom severity. Multivariate analyses of variance (MANOVAs) with covariates were conducted to explore the relationship between subdimensions of metacognitive beliefs and OCD disease status. In addition, for the OCD patients, Pearson's correlation was performed between baseline MCQ subdimensions and Obsessive–Compulsive Inventory-Revised-Korean subscales (OCI-R-K). Finally, in a subset of drug-free OCD patients (*n* = 144), the MCQ was reassessed after 3 months of treatment and patterns of changes in subdimensions of the MCQ were examined.

**Results:** Patients with OCD scored significantly higher on the four dimensions of the MCQ. There were significant associations between all MCQ subdimensions and OCI-R-K subscales. In the repeated-measure MANOVA, a significant group (non-responders vs. responders)-by-time interaction effect was found only for the negative beliefs about the uncontrollability and danger of worry (NB) subdimension (*F* = 10.75; η^2^ = 0.072; *p* = 0.001).

**Conclusion:** The presence of dysfunctional metacognitive beliefs in OCD subjects and their association with OCD characteristics suggest that dysfunctional metacognitions may play a crucial role in the pathophysiology of OCD. Improvement of metacognitive beliefs in the NB dimension may be a clinically meaningful correlate of good treatment response in the pharmacological treatment of OCD.

## Introduction

Obsessive–compulsive disorder (OCD) is a debilitating psychiatric disorder characterized by obsessions, which are intrusive thoughts and images that increase anxiety, and compulsions, which are repetitive or ritualistic actions to decrease anxiety (1). Metacognition, the knowledge or beliefs about thinking and strategies used to control thinking processes, has been suggested to play an important role in the maintenance of such symptoms (2). The first metacognitive model of OCD proposed by Wells assumes that beliefs about the importance of thinking cause a person to assign high significance to his/her thoughts, and when that person experiences an obsession, metacognitive processing is activated, which accesses knowledge about the intrusion (3). The metacognitive model of OCD has been validated with empirical studies. In a study of 238 University students, thought-fusion beliefs, beliefs about the need to perform rituals, and criteria that signal rituals can be stopped explained the variance in obsessive–compulsive symptoms in the theorized causal sequence (4). In another study of 304 community volunteers, thought-fusion beliefs and beliefs about rituals predicted obsessive-compulsive symptoms, further providing empirical support for the metacognitive model (5). In addition, metacognitive therapy (MCT), which specifically focuses on metacognition, has also been developed and has shown promising results (6). In a study of 25 OCD patients, after 15 weekly sessions of MCT, 74% of patients met the criteria for recovery (7). In another study that compared MCT to OCD CBT, 95 OCD patients who participated in 12 weekly sessions of MCT improved significantly more than the CBT cohort; 86.3% of patients in the MCT group responded compared with 64% in the CBT group (8).

There has been a series of studies that examined the relationship between metacognition and OC symptoms in the general population. In a study of 120 undergraduate students, there was a positive association between metacognitive factors and OC symptoms (2). In another study of 110 undergraduate students, metacognitions as measured by the Metacognitions Questionnaire (MCQ) were significant predictors of OC symptoms such as symmetry and unacceptable thoughts (9). Finally, in a study of 305 community volunteers, metacognitive beliefs were found to be related to obsessive–compulsive behavior such as washing and checking (10).

Although the relationship between metacognition and OCD has been extensively studied on a conceptual level, there is still a lack of clinical data regarding the subject matter. There are a few studies involving OCD patients, but they have been limited by small sample size, with most studies involving fewer than 100 OCD patients. In a study of 114 OCD patients using the MCQ, OCD patients scored significantly higher than controls on two dimensions: negative beliefs about worry concerning uncontrollability and danger, and beliefs about the need to control thoughts (11). Similarly, in a study of 117 subjects (52 OCD patients and 65 healthy controls) and a study of 97 subjects (51 OCD patients and 46 healthy controls), the same two sub-dimensions of the MCQ were elevated in OCD patients compared to controls (12). In a study of 75 OCD patients, OCD patients reported significantly more positive beliefs about rituals and stop signals than did the patients with anxiety disorder, patients with depressive disorder, and non-clinical controls (13). Finally, in a study of 51 OCD patients and 46 controls, negative beliefs about worry concerning uncontrollability and danger were shown to be significantly higher in OCD patients (14). In addition to the small sample size mentioned above, some studies were also uncontrolled for depression and anxiety, which are known to affect metacognition (12).

There is also a lack of studies that examined the change in metacognition in OCD patients as they receive treatment. In one of those studies, Solem et al. (15) followed 83 OCD patients who received 12 weekly sessions of exposure and response prevention (ERP). After ERP, there was a significant decrease in the Y-BOCS and MCQ scores, and regression analysis showed that a change in metacognition explained 22% of the variance in OCD symptoms (15). To our knowledge, no study has observed changes in metacognition in OCD patients receiving pharmacological treatment. In our prior study of 132 OCD patients, the metacognition subdimension of positive beliefs about worry at baseline was a significant predictor of early symptom improvement after 1 month of pharmacotherapy, but metacognition was not measured repeatedly (16).

The present study aimed to examine how metacognitive beliefs are associated with the diagnostic status and subtypes of OCD and OCD-related characteristics. In addition, we examined the pattern of changes in the subdimensions of metacognitive beliefs according to treatment response by assessing the metacognitive beliefs at baseline and repeating the measurement following 3 months of pharmacological treatment.

## Methods

### Participants

A total of 562 OCD patients and 236 controls were recruited from the outpatient clinic of Severance Hospital in Seoul, Korea, through consecutive screening and enrollment from 2010 to 2020. All participants were assessed using the Structured Clinical Interview for DSM-IV Axis I disorders (SCID-I) by trained psychiatrists. Of the 562 OCD patients, 144 patients were drug-naïve or drug-free of any psychiatric medication for at least 3 months prior to participation. These patients were started on pharmacological treatment depending on the clinical decision of a psychiatrist who was independent from the rater and statistical analyst in this study. All participants provided written informed consent prior to the beginning of this study. The study protocol was approved by the Institutional Review Board of Severance Hospital, and all methods of this study were carried out in accordance with the approved guidelines. The IRB approval numbers were 4-2010-0577 and 4-2015-0655.

### Assessment

At baseline, all participants were asked to answer standardized questions on social-demographic characteristics including age, sex, year of education, marital status, occupation, average monthly income, height/weight, age of OCD onset (for patients only), and psychiatric medication. All controls were never diagnosed with psychiatric illness and were drug-naïve. At baseline, all participants were also asked to answer the following standardized questionnaires: the Yale–Brown Obsessive Compulsive Scale (Y-BOCS), Montgomery–Asberg Depression Rating Scale (MADRS), and Obsessive–Compulsive Inventory-Revised-Korean (OCI-R-K). To measure each patient's metacognitive beliefs, the MCQ was completed.

The 144 drug-free patients who started on pharmacological treatment were again assessed after 3 months. At the 3-month follow-up, the participants completed the same questionnaires that they completed at baseline.

## Clinical Characteristics

### Yale–Brown Obsessive Compulsive Scale

To evaluate the severity of obsessive–compulsive disorder symptoms, the Y-BOCS was administered to OCD patients at baseline and at 3-month follow-up. The Y-BOCS is a 10-item scale, and each item is scored on a scale ranging from 0 to 4, with higher scores indicating higher symptom severity (17).

### Obsessive-Compulsive Inventory-Revised-Korean

The OCI-R-K is another scale that evaluates the severity of obsessive–compulsive disorder symptoms (18). It is an 18-item scale, and each item is scored on a scale ranging from 0 to 4, with higher scores indicating higher symptom severity. It comprises six subscales of OCD symptoms: washing, checking, ordering, obsessing, hoarding, and neutralizing.

### Montgomery–Asberg Depression Rating Scale

To evaluate the severity of depressive symptoms, the MADRS was administered to both cases and controls at baseline and at the 3-month follow-up. MADRS is a 10-item scale, and each item is scored on a scale ranging from 0 to 6, with higher scores indicating higher depressive symptom severity (19).

## Metacognitive Beliefs

### Metacognitions Questionnaire

The MCQ was developed to measure beliefs about worry and intrusions (20). It is composed of 65 questions, which are scored on a four-point scale ranging from 1 (“Do not agree”) to 4 (“Agree very much”). The MCQ is composed of five subscales: positive beliefs about worrying (PB), negative beliefs about the uncontrollability and danger of worry (NB), beliefs about the need for control of thoughts (NFC), beliefs concerning cognitive competence (CC), and cognitive self-consciousness (CSC). The MCQ was administered to both cases and controls at baseline, and to cases at the 3-month follow-up.

### Statistical Analysis

Statistical analysis was conducted using Statistical Package for the Social Sciences version 25.0 (SPSS Inc., Chicago, IL, USA). Missing value analysis was performed using the SPSS expectation-maximization (EM) algorithm. The EM imputation provides unbiased parameter estimates when data are missing completely at random and a very small portion of data are missing (21). Subjects were excluded from the analysis if missing data exceeded 5% of the total number of items, and missing values below 5% were imputed using the EM estimates. Independent sample *t*-test and chi-square test were performed to compare the demographic information between OCD patients and controls. Multivariate analysis of variance (MANOVA) was performed to compare baseline MCQ scores between OCD patients and controls, while controlling for age, sex, and MADRS. For OCD patients, Pearson's correlation was performed to observe the correlation of baseline MCQ subdimensions with OCI-R-K subscales and the Y-BOCS. For OCD patients who were followed up after 3 months, repeated measures ANOVA was performed, controlling for age, sex, and MADRS; treatment response was set as the between-subjects factor and time was set as the within-subjects factor, to test for the difference in MCQ subdimension scores between baseline and follow-up. Response was defined as a ≥35% reduction in the Y-BOCS at follow-up (22).

## Results

### Demographic and Clinical Characteristics

The demographic and clinical characteristics of the patients are presented in Table 1. There was no difference in age, sex, or duration of education between OCD patients and controls, but OCD patients scored significantly higher on the Y-BOCS [25.52 (7.01) vs. 0.47 (1.86), *p* < 0.001] and MADRS [20.45 (9.64) vs. 3.39 (4.09), *p* < 0.001] compared to controls. All patients on follow-up were taking serotonin reuptake inhibitors (SRIs), including escitalopram (*n* = 108), and some of them were taking concomitant medications including benzodiazepines (*n* = 44) and atypical antipsychotics (*n* = 26).

**Table 1:** Demographic and clinical characteristics of participants.

	**OCD (*n =* 562)**	**Control (*n =* 236)**	***p***
**Age (years)**	28.12 (8.25)	27.47 (8.14)	0.521
**Sex, male, %**	61.03%	58.05%	0.432
**Education (years)**	13.78 (2.35)	13.95 (2.20)	0.151
**Y-BOCS**	25.52 (7.01)	0.47 (1.86)	**<0.001**
**Onset of illness (years)**	18.12 (7.66)	NA	NA
**Duration of illness (years)**	10.92 (8.49)	NA	NA
**MADRS**	20.45 (9.64)	3.39 (4.09)	**<0.001**

*The means (standard deviations) are given for age, education, Y-BOCS, Onset of illness, Duration of illness, and MADRS*.

*OCD, obsessive compulsive disorder; MADRS, Montgomery–Åsberg Depression Rating Scale; Y-BOCS, Yale–Brown Obsessive Compulsive Scale*.

*P-values in bold are significant at α < 0.05*.

### Comparison of MCQ Scores Between Patients and Controls

The comparison of MCQ scores between OCD patients and controls are presented in Table 2. When uncontrolled, there was a significant difference in the NB (*p* < 0.001), NFC (*p* < 0.001), CC (*p* < 0.001), and CSC (*p* < 0.001) subdimensions, as well as in the total MCQ score (*p* < 0.001). When the analysis was controlled for age, sex, and MADRS, the results were similar as there was still a significant difference in NB (*p* < 0.001), NFC (*p* = 0.003), CC (*p* < 0.001), and CSC (*p* = 0.001) subdimensions as well as in the total MCQ score (*p* < 0.001) between OCD patients and controls.

**Table 2:** Comparison of MCQ scores between patients and controls.

**MCQ**	**OCD (*n =* 562)**	**Control (*n =* 236)**	**Uncontrolled**		**Controlled for age, sex, MADRS**		
			***t***	***p***	***F***	**$\eta_{\text{p}}^{2}$**	***p***
**PB**	37.67 (11.28)	36.94 (9.01)	−0.97	0.333	1.65	0.002	0.200
**NB**	46.53 (11.65)	25.78 (7.21)	−30.52	**<0.001**	155.99	0.176	**<0.001**
**NFC**	20.23 (6.40)	14.64 (4.33)	−14.30	**<0.001**	9.12	0.012	**0.003**
**CC**	31.72 (8.24)	21.02 (4.78)	−22.92	**<0.001**	56.67	0.072	**<0.001**
**CSC**	20.44 (4.52)	17.12 (3.74)	−10.75	**<0.001**	11.47	0.015	**0.001**
**Total**	156.59 (31.47)	115.45 (21.27)	−21.41	**<0.001**	45.04	0.068	**<0.001**

*Pillai's trace = 0.67; F = 296.78; p < 0.001. MCQ, metacognitions questionnaire; OCD, obsessive compulsive disorder; MADRS, Montgomery–Åsberg Depression Rating Scale; PB, positive beliefs about worry; NB, negative beliefs about the uncontrollability and danger of worry; NFC, beliefs about the need for control of thoughts; CC, beliefs concerning cognitive competence; CSC, cognitive self-consciousness*.

*P-values in bold are significant at α < 0.05*.

### Correlation Between MCQ, OCI-R-K, and Y-BOCS Scores

The correlations between the MCQ subdimensions, OCI-R-K subscales, and Y-BOCS scores are presented in Table 3. There were significant associations between all MCQ subdimensions and OCI-R-K subscales.

**Table 3:** Correlation between MCQ subdimensions, OCI-R-K subscales, and Y-BOCS scores.

**MCQ**	**Hoarding**	**Washing**	**Checking**	**Neutralizing**	**Ordering**	**Obsessing**	**Total**	**YBOCS**
**PB**	0.224^**^	0.160^**^	0.261^**^	0.273^**^	0.302^**^	0.164^**^	0.331^**^	0.104^*^
**NB**	0.259^**^	0.090^*^	0.259^**^	0.243^**^	0.153^**^	0.543^**^	0.358^**^	0.234^**^
**NFC**	0.280^**^	0.170^**^	0.248^**^	0.217^**^	0.207^**^	0.398^**^	0.344^**^	0.229^**^
**CC**	0.343^**^	0.144^**^	0.216^**^	0.323^**^	0.236^**^	0.516^**^	0.407^**^	0.168^**^
**CSC**	0.276^**^	0.113^**^	0.293^**^	0.206^**^	0.240^**^	0.399^**^	0.374^**^	0.185^**^
**Total**	0.363^**^	0.179^**^	0.339^**^	0.347^**^	0.304^**^	0.533^**^	0.482^**^	0.241^**^

* *p < 0.01*,

** *p < 0.001*,

*MCQ, metacognitions questionnaire; PB, positive beliefs about worry; NB, negative beliefs about the uncontrollability and danger of worry; NFC, beliefs about the need for control of thoughts; CC, beliefs concerning cognitive competence; CSC, cognitive self-consciousness; Y-BOCS, Yale–Brown Obsessive Compulsive Scale; OCI-R-K, Obsessive Compulsive Inventory-Revised-Korean*.

*Pearson's correlation coefficient larger than 0.3 is presented in bold*.

### MCQ Score at the 3-Month Follow-Up

**Figure 1:**
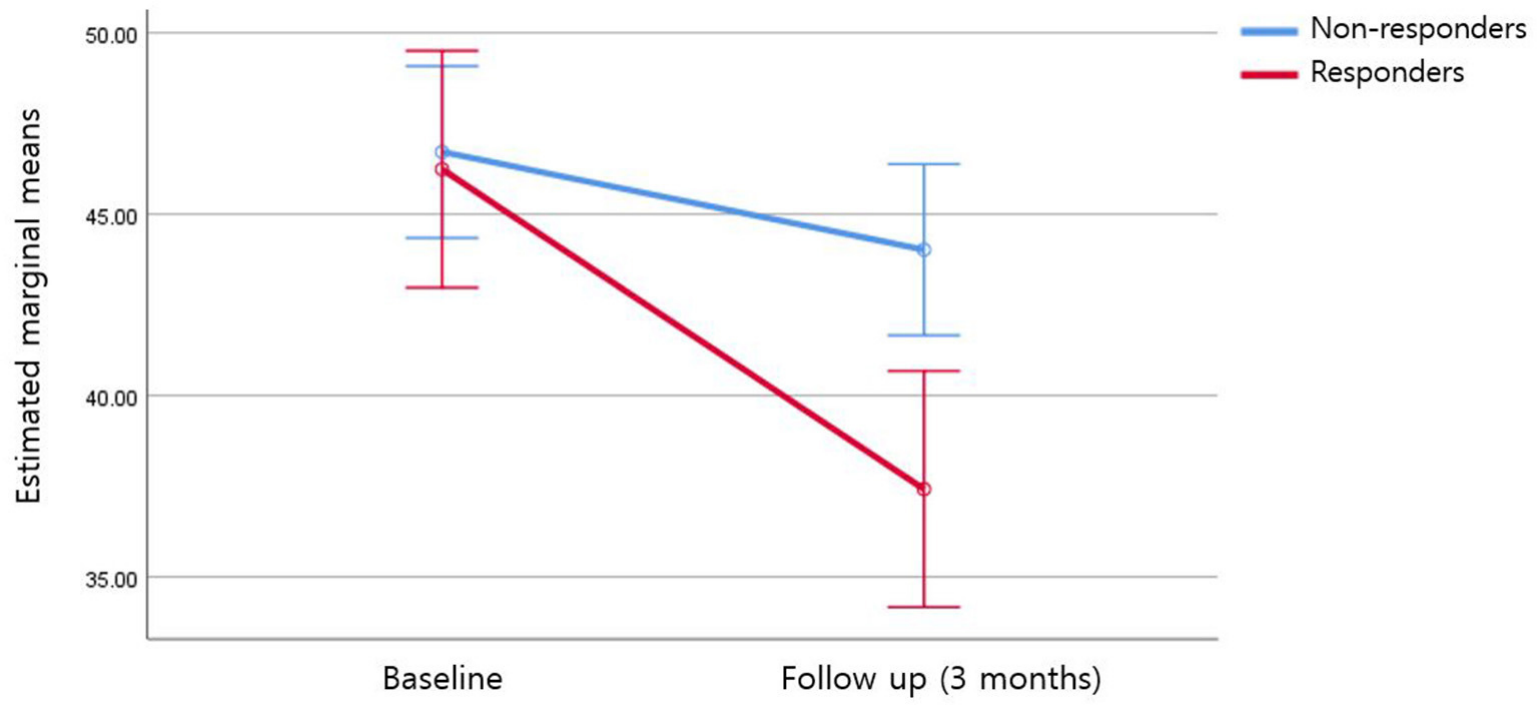
Negative beliefs about the uncontrollability and danger of worry at follow-up.

At follow-up, 48 patients (33.3%) showed ≥35% reduction in the Y-BOCS and were classified as responders, and 96 (66.7%) were non-responders. The Y-BOCS and MADRS scores of the patients on follow-up are presented in Table 4. The analysis revealed a statistically significant group-by-time interaction for the NB subdimension (*F* = 10.75; $\eta_{\text{p}}^{2}$ = 0.072; *p* = 0.001) and total MCQ score (*F* = 5.364; $\eta_{\text{p}}^{2}$ = 0.037; *p* = 0.022) controlling for age, sex, and MADRS (Figure 1, Table 4). There was no significant group-by-time interaction effect for other MCQ subdimensions, and there was no main effect of time (Table 4).

**Table 4:** MCQ scores at baseline and follow-up.

**MCQ**	**Baseline**		**Follow-up (3 months)**		**Factor time**		**Factor group**		**Time x group interaction**	
	**Non-responders**	**Responders**	**Non-responders**	**Responders**	***F***	***P***	***F***	***P***	***F***	***P***
**PB**	37.18 ± 11.34	37.50 ± 10.78	40.70 ± 12.80	39.37 ± 11.22	0.345	0.558	0.020	0.887	1.076	0.302
**NB**	46.39 ± 12.29	44.81 ± 11.44	43.98 ± 11.25	36.28 ± 11.78	0.020	0.889	3.938	**0.049**	10.757	**0.001**
**NFC**	20.46 ± 6.42	18.49 ± 5.49	20.95 ± 6.57	18.32 ± 5.49	3.373	0.068	4.157	**0.043**	0.546	0.461
**CC**	31.69 ± 8.84	29.12 ± 6.68	30.52 ± 8.65	26.89 ± 8.47	0.185	0.667	3.277	0.072	0.974	0.325
**CSC**	20.25 ± 4.97	19.47 ± 4.28	20.46 ± 4.43	18.28 ± 3.39	0.000	0.988	2.421	0.122	3.308	0.071
**Total**	156.48 ± 34.06	150.06 ± 26.23	156.54 ± 33.62	139.34 ± 30.50	0.131	0.718	3.53	0.062	5.364	**0.022**
Y-BOCS	27.18 ± 5.37	28.71 ± 6.11	25.04 ± 5.36	13.19 ± 5.04	NA					
MADRS	19.76 ± 9.17	21.09 ± 8.06	18.17 ± 9.12	8.88 ± 6.36						

*Pillai's trace = 0.63; F = 45.97; p < 0.001. MCQ, metacognitions questionnaire; PB, positive beliefs about worry; NB, negative beliefs about the uncontrollability and danger of worry; NFC, beliefs about the need for control of thoughts; CC, beliefs concerning cognitive competence; CSC, cognitive self-consciousness; Y-BOCS, Yale-Brown Obsessive Compulsive Scale; MADRS, Montgomery-Asberg Depression Rating Scale*.

*P-values in bold are significant at α < 0.05*.

## Discussion

In the present study, we adopted both a cross-sectional and a prospective perspective to examine the role of dysfunctional metacognitive beliefs in OCD. The results showed that compared to healthy controls, OCD patients scored significantly higher on subdimensions of negative beliefs about the uncontrollability and danger of worry (NB), beliefs about the need for control of thoughts (NFC), cognitive self-consciousness (CSC), and beliefs concerning cognitive competence (CC). In addition, when changes in metacognitive beliefs were observed prospectively as the patients received pharmacological treatment, there was a significant group (responders vs. non-responders)-by-time interaction effect in the NB subdimension. The results indicate that certain subdimensions of metacognition may play a crucial role in the pathophysiology and clinical course of OCD.

Our comparisons of metacognitive beliefs between OCD patients and healthy controls at a cross-sectional level revealed that OCD patients scored higher on the subdimensions of NB, NFC, CSC, and CC, in both uncontrolled and controlled analyses. Moreover, in the correlational analysis, these four dimensions were associated with all of the OCD symptom dimensions. Notably, NB was the subdimension with the largest difference between patients and controls. This result is consistent with findings shown in previous reports of OCD with smaller samples using the MCQ30, a shorter version of the original MCQ (12, 14). In one study, the authors found NB to be the most successful subdimension in differentiating individuals with OCD from controls. In addition, regarding the other subdimensions (NFC, CSC, and CC), although the results have been inconsistent in previous studies with smaller sample sizes (12, 14), our study found that NFC, CSC, and CC were all significantly elevated in patients with OCD. It is possible that prior studies might have been underpowered to detect the actual differences in those dimensions. High metacognitive beliefs in specific dimensions, such as negative beliefs about the uncontrollability of thoughts and danger, which relate to the perceived dangerousness of thoughts (e.g., “Worrying is dangerous for me”) and beliefs about need to control thought (e.g., “I should be in control of my thoughts all of the time”), may contribute to the excessive effort of suppressing normally occurring intrusive thoughts by perceiving them as uncontrollable and dangerous and thus converting them into significant, unwanted obsessions and pathologic rituals. This explanation can be supported by Wegner's Ironic Process Theory of mental control, which posits that attempts to suppress intrusive thoughts are counterproductive as shown in the “white bear” study of the paradoxical effect of thought suppression (23). In a meta-analytic review of dysfunctional metacognition in different mental disorders, NB and NFC showed the largest effect sizes out of the five MCQ subdimensions (Hedges' *g* = 1.5417 and 1.643, respectively) when comparing OCD patients to controls (24).

In addition, our prospective results showed that there was a significant interaction effect of time and group (responders vs. non-responders) only for the subdimension of NB following 3 months of pharmacological treatment. This finding indicates that at least early on in the course of pharmacotherapy, a decrease in NB is associated with an improvement in OC symptoms. These results also imply that metacognitive therapies that target negative beliefs about the uncontrollability and danger of worry may be helpful in controlling OC symptoms in OCD patients. Further studies are needed to confirm which subdimensions of metacognition change according to time and treatment.

Notably, the subdimension of PB showed a different pattern from other subdimensions. While PB was not associated with OCD status in the between-group analysis, the pattern of association with OCD symptom dimensions was different for PB than for other MCQ subdimensions. For the ordering symptom dimension (which corresponds to symmetry/order-related obsessions and compulsions), PB showed the strongest association out of all MCQ subdimensions, while for the obsessing symptom dimension [which corresponds to sexual/religious (forbidden/taboo) thoughts], it showed the weakest association. In our preliminary report, OCD patients with higher PB baseline scores were associated with poorer outcomes in terms of early improvement after 1 month of pharmacotherapy (16). Although the role of PB in OCD has not been extensively studied, it has been examined in other psychiatric disorders. In a study that examined metacognitive beliefs in people with psychotic disorders, positive beliefs about psychotic symptoms (e.g., suspiciousness is good and keeps an individual safe) were suggested to contribute to more frequent and severe psychotic symptoms (25). In a study that examined the role of metacognition in depression, the results suggest that positive metacognitive beliefs may serve as a trigger for the application of rumination as an incompatible coping mechanism for depression (26). These findings suggest that PB is a subdimension that works as a psychological trait that affects the disease course by reinforcing maladaptive coping with the psychopathology of OCD by considering compulsive behavior such as arranging and organizing as safe and protective rituals.

There are some study limitations that should be noted. First, the follow-up period was only 3 months, which may not be enough to observe response or remission, since OCD is a chronic condition. Although 3 months may be a suitable period for follow-up in the short term because patients with OCD typically take 10–12 weeks to respond to SRIs (27), future longitudinal studies with more than 1 year of follow-up are needed to see how certain subdimensions of metacognition change as OCD patients are treated with pharmacotherapy. Studies with longer follow-up will also be helpful in observing whether the change in metacognition is permanent or only temporary. Second, we did not consider other measures of cognitive function such as working memory capacity, which may affect metacognition (28). Third, because the subjects of our study were OCD patients who were referred to university hospitals, most of the patients showed severe OC symptoms, which may not be representative of OCD patients in general. Fourth, we did not control for the different types of psychiatric medication that the drug-naïve patients started taking from baseline; different psychiatric medications may affect OC symptom severity differently. Fifth, some of the scales used in this study including the MCQ are self-reported, and self-report may produce answers that are exaggerated and affected by various biases such as social desirability bias. Sixth, we did not control for other clinical characteristics such as comorbid Axis II personality disorder, which could affect treatment response (29). Finally, we did not include any OCD-specific measure of metacognition. While the MCQ is a reliable and valid measure of metacognitions, it is not an OCD-specific measure (20).

In conclusion, this study showed that compared to healthy controls, metacognitive subdimensions of NB, NFC, CC, and CSC were elevated in OCD patients, and when the patients are treated over time, a decrease in certain dimensions, specifically NB, was observed in responders, but not in non-responders. Understanding specific metacognitive difficulties and tailoring metacognitive interventions could be helpful in enhancing symptom improvement and treatment response for patients with OCD. Future longitudinal studies with longer follow-up are needed to establish how metacognitive beliefs are involved in the pathophysiology of OCD and how certain metacognitive subdimensions change in the long term as OC symptoms are treated.

## Data Availability Statement

The raw data supporting the conclusions of this article will be made available by the authors, without undue reservation.

## Ethics Statement

The studies involving human participants were reviewed and approved by Institutional Review Board of Severance Hospital. The patients/participants provided their written informed consent to participate in this study.

## Author Contributions

SJK and JIK conceived and planned the study. SJK and SJ contributed to data collection and management. STK, SJK, and JIK undertook the statistical analyses and interpreted the findings. STK and JIK wrote the manuscript. CIP and HWK provided scientific input and helped edit the manuscript. All authors contributed to and have approved the final manuscript.

## Conflict of Interest

The authors declare that the research was conducted in the absence of any commercial or financial relationships that could be construed as a potential conflict of interest.
